# Advancing the sleep/wake schedule impacts the sleep of African-Americans more than European-Americans

**DOI:** 10.1371/journal.pone.0186887

**Published:** 2017-10-23

**Authors:** Gemma M. Paech, Stephanie J. Crowley, Louis F. Fogg, Charmane I. Eastman

**Affiliations:** Biological Rhythms Research Laboratory, Department of Behavioral Sciences, Rush University Medical Center, Chicago, Illinois, United States of America; Universita degli Studi di Bologna, ITALY

## Abstract

There are differences in sleep duration between Blacks/African-Americans and Whites/European-Americans. Recently, we found differences between these ancestry groups in the circadian system, such as circadian period and the magnitude of phase shifts. Here we document the role of ancestry on sleep and cognitive performance before and after a 9-h advance in the sleep/wake schedule similar to flying east or having a large advance in sleep times due to shiftwork, both of which produce extreme circadian misalignment. Non-Hispanic African and European-Americans (N = 20 and 17 respectively, aged 21–43 years) were scheduled to four baseline days each with 8 h time in bed based on their habitual sleep schedule. This sleep/wake schedule was then advanced 9 h earlier for three days. Sleep was monitored using actigraphy. During the last two baseline/aligned days and the first two advanced/misaligned days, beginning 2 h after waking, cognitive performance was measured every 3 h using the Automated Neuropsychological Assessment Metrics (ANAM) test battery. Mixed model ANOVAs assessed the effects of ancestry (African-American or European-American) and condition (baseline/aligned or advanced/misaligned) on sleep and cognitive performance. There was decreased sleep and impaired performance in both ancestry groups during the advanced/misaligned days compared to the baseline/aligned days. In addition, African-Americans obtained less sleep than European-Americans, especially on the first two days of circadian misalignment. Cognitive performance did not differ between African-Americans and European-Americans during baseline days. During the two advanced/misaligned days, however, African-Americans tended to perform slightly worse compared to European-Americans, particularly at times corresponding to the end of the baseline sleep episodes. Advancing the sleep/wake schedule, creating extreme circadian misalignment, had a greater impact on the sleep of African-Americans than European-Americans. Ancestry differences in sleep appear to be exacerbated when the sleep/wake schedule is advanced, which may have implications for individuals undertaking shiftwork and transmeridian travel.

## Introduction

There is increasing evidence that there are differences in sleep duration between groups with different evolutionary ancestry [1, 2]. Population-based survey studies have shown that Blacks/African-Americans are more likely to report obtaining less sleep compared to Whites/European-Americans [3–8]. Studies utilizing objective measures of sleep, such as actigraphy and polysomnography (PSG), have also demonstrated that African-Americans obtain less sleep compared to European-Americans [9–15]. There is, however, some contradicting evidence suggesting that there are no such differences in sleep duration [16, 17], or that African-Americans may obtain more sleep than European-Americans [18]. Despite these inconsistencies, which may be attributed to variances in participant demographics, it is clear that there are differences in sleep duration, with most research suggesting that African-Americans obtain less sleep than European-Americans.

Although researchers have investigated differences in overall sleep duration according to ethnic/ancestral history, there is, to our knowledge, no research on how these differences may be impacted by shifting the sleep/wake schedule, similar to that experienced with shift work or jet travel. Sleep duration is influenced by the endogenous circadian timing system such that sleep is typically shortest when the sleep episode is misaligned relative to endogenous circadian rhythms [19–22]. Repeated evidence from our laboratory shows that there are differences between African-Americans and European-Americans in the circadian timing system, such as a shorter free-running circadian period in African-Americans [23–27]. More recent evidence from our laboratory demonstrates that there are differences between African-Americans and European-Americans in the way the circadian timing system phase shifts in response to a large abrupt advance shift in the sleep/wake (and light-dark) schedule [23, 26]. Therefore, shifting the sleep/wake schedule may affect the sleep of African-Americans differently than European-Americans.

The shorter sleep experienced by African-Americans under normal circumstances [3, 5, 6] may subsequently result in cognitive performance impairments such as decreased reaction time, increased sleepiness, and mood, especially if sleep is restricted over several days [28–30]. Cognitive performance is also influenced by the endogenous circadian timing system, with cognitive performance impairments observed during circadian misalignment, when the sleep/wake schedule is misaligned relative to endogenous circadian rhythms [29, 31–34]. The circadian influence on cognitive performance is further exacerbated when sleep is restricted [35–37]. Although there has been extensive research into the effects of shifted sleep on cognitive performance [38–45], it is unknown if shifting the sleep/wake schedule has different effects on performance in African-Americans compared to European-Americans.

In a recent study [23], we measured the free-running circadian period (τ) of non-Hispanic African-Americans and non-Hispanic European-Americans. In addition, the sleep/wake (light/dark) schedule was shifted earlier by 9-h (i.e., advanced), similar to flying east across 9 time zones (e.g., Chicago to Kenya) or having a large advance in sleep times due to shift work. Results on the relationship between the circadian period and the phase shift have been reported [23]. Following 3 days of the shifted sleep/wake schedule, no individual phase shifted enough to even come close to complete re-entrainment. Thus, there was extreme circadian misalignment; the sleep/wake schedule was misaligned relative to endogenous circadian rhythms [23]. The current analysis presents results of sleep, cognitive performance, sleepiness and mood during baseline/aligned days (with sleep according to Chicago time) and advanced/misaligned days (with sleep according to Kenya time). We hypothesized that African-Americans would obtain more sleep and have better cognitive performance compared to European-Americans during advanced/misaligned days, as a shorter circadian period facilitates phase advances [23, 26].

## Materials and methods

### Participants

Participants were recruited with on-line ads and flyers from November 2012 through April 2014. An initial questionnaire excluded a majority of these individuals as they did not meet inclusion criteria: having a BMI less than 35, being a non-smoker, not being Hispanic or Latino and both biological parents being either Black/African-American or White/European-American. Follow up phone calls and in person interviews excluded other participants who did not meet more stringent inclusion criteria such as a clean drug screen, and no reported health or sleep issues. We did not keep track of how many people applied to be in the study. There were 42 people who were enrolled and who signed consent forms three to four days before the start of the 14-day laboratory study which occurred between January 2013 and May 2014. Of these, 39 started the 14-day study, 38 completed the study and 37 were included in the current analyses. Data from one participant (male) was excluded as ancestry DNA results (see below) indicated he was 46% Sub-Saharan African and 49% European and therefore could not be included in the African-American group or the European-American group. The 37 participants were between 21 and 43 years old.

Participants completed a Family/Ancestor Questionnaire (S1 Appendix) which asked participants to check all of the following categories that applied to them, their biological parents, and all four grandparents: White, Black or African-American, Asian, Hispanic or Latino, European, Middle Eastern, Far East Asian, Indian Subcontinent, North African, Afro-Caribbean, American Indian or Alaska native, Native Hawaiian or other Pacific Islander, Other, Don’t Know. Participants self-identified as being either African-American (n = 20) or white (n = 17). None of the participants self-identified as being “Hispanic or Latino.”

DNA samples were collected from Buccal (cheek) swabs and analyzed (AncestrybyDNA, DNA Diagnostics Center, Fairfield, OH) to confirm self-reported ancestry. This company performed biogeographical ancestry estimates based on 176 ancestry informative markers, also known as population-specific alleles, which show large frequency differences between populations [46, 47]. Results were returned several weeks later with percentages for each subject in four categories: European, Sub-Saharan African, East Asian and Indigenous American (See the first table in [23]).

Participants completed the Morningness-Eveningness Questionnaire (MEQ) [48] and the Munich Chronotype Questionnaire (MCTQ) [49]. According to the MEQ, there were eight morning types and one evening type in the African-American group and three morning types and no evening types in the European-American group.

Participants also assessed their subjective socioeconomic status (SES) using a socioeconomic status ladder [50], which consists of a drawing of a 10 rung ladder. The top of the ladder represents those with the most money, most education and best jobs, and the bottom represents those who have the least money, least education and worst or no job. Participants were instructed to place themselves on the ladder in terms of where they think they stood. This position was then translated into a score between 1 and 10, where 1 = bottom rung (i.e., lowest SES) and 10 = top rung (i.e., highest SES). In this way, higher scores are representative of a higher perceived SES. Participant demographics are shown in Table 1. There were no differences (as determined by independent t-tests; multiple comparison corrections were not applied) in participant demographics (Table 1).

**Table 1:** Participant demographics.

	Combined	African-American	European-American
N	37	20	17
Sex	17 F, 20 M	45% F	47% F
Age	30.97 ± 6.74	32.10 ± 7.46	29.65 ± 5.71
BMI	24.79 ± 4.27	24.95 ± 4.63	24.61 ± 3.92
SES	5.76 ± 1.42	5.60 ± 1.31	5.94 ± 1.56
MEQ	53.16 ± 7.77	54.50 ± 9.36	51.59 ± 5.20
MSF	5.15 ± 1.38	5.14 ± 1.63	5.17 ± 1.07
Bedtime	00:12 ± 1:23	00:03 ± 01:38	00:24 ± 01:03
Wake-time	08:12 ± 01:23	08:03 ± 01:38	08:24 ± 01:03

Values shown as mean ± SD.

BMI: Body Mass Index. SES: Subjective Socioeconomic Score. MEQ: Morningness-Eveningness Questionnaire. MSF: Mid Sleep on Free days from Munich Chronotype Questionnaire (MCTQ). Bedtime: scheduled baseline bedtime. Wake-time: scheduled baseline wake-time, 8 h after bedtime. Independent t-tests showed no differences between African-Americans and European-Americans for any of the demographics.

All participants were physically and psychologically healthy as determined by a Health Information Questionnaire (S2 Appendix), a For Women Only Questionnaire (S3 Appendix), the Beck Depression Inventory [51] and the Minnesota Multiphasic Personality Inventory (MMPI-2) [52]. Participants also completed the Pittsburg Sleep Quality Index (PSQI) [53], the Epworth Sleepiness Scale (ESS) [54], the Berlin Questionnaire [55] and the Insomnia Severity Index [56] to determine if they had sleep disturbances.

Participants were all non-smokers and free from medication, except for 4 women (2 African-Americans and 2 European-Americans) using oral contraceptives. Participants were low to moderate consumers of caffeine (≤ 300mg caffeine/day; equivalent to < 2cups coffee/day) and alcohol (≤ 3 standard drinks/day). Participants had not undertaken any night shift work in the month prior to the study. Participants were asked to abstain from caffeine in the four days preceding the study.

The study had approval from the Rush University Medical Center Institutional Review Board, in accordance with standards set by the Declaration of Helsinki. All participants gave written informed consent to participant in the study and were made aware that participation was completely voluntary and that they could withdraw at any time. Upon completion participants were given a financial compensation for their participation.

### Study design

**Fig 1:**
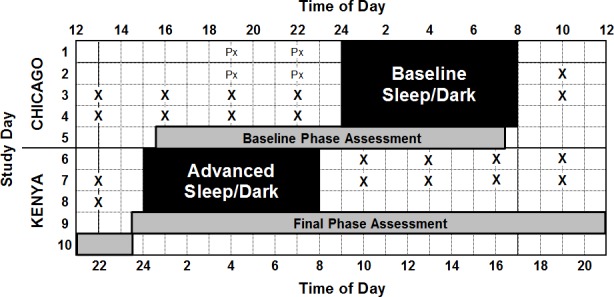
Protocol diagram. Time of day at the top is Chicago time, and time of day on the bottom is Kenyan time (9-h later than Chicago time). Study day is shown on the left. Black shading shows timing of scheduled sleep periods. Schedules were individualized for each participant to best match their habitual sleep times. This diagram shows the protocol for a participant on a 00:00–08:00 baseline sleep schedule. Days 1–4 were baseline during which participants remained on local, Chicago time (as indicated on the left). On days 6–9 the sleep schedule was shifted 9-h earlier (advanced) as though participants had traveled to Kenya, and the wall clocks in the bedrooms were changed to indicate the shifted, Kenyan time. During baseline days the sleep schedule was aligned with each participant’s circadian rhythms, whereas during advanced days the sleep schedule was misaligned. “X” shows the timing of the Automated Neuropsychological Assessment Metrics (ANAM) performance battery and “Px” shows the timing of practice ANAM tests. Tests were given relative to each participants scheduled sleep times; 2 h after waking, and then every 3 h with a total of 5 tests per day. Light grey shading shows the timing of circadian phase assessments, during which the dim light melatonin onset (DLMO) was assessed.

The current study took place at the Biological Rhythms Research Laboratory in Chicago, USA. Participants completed the protocol in groups of two or three, usually with a mixture of African- and European-Americans in each group. The current report focuses on the last 10 days (labeled here as Days 1–10, Fig 1) of a larger 14-day study [23]. To see the protocol diagram for the full 14 days, see Eastman et al. [23]. With the exception of one 8-h break after the first baseline sleep episode (Day 1, Fig 1), participants remained in the laboratory for all 14 days of the study and were continuously monitored by research staff. Caffeine and alcohol were not permitted while in the laboratory. During the 8-h break, participants were reminded not to consume alcohol or caffeine, or take any naps. Upon re-entry to the laboratory participants were given a urine drug screen and were breathalyzed. While participants were not required to leave the laboratory, a majority of participants chose to do so.

Participants were scheduled to four baseline days with 8h time in bed (TIB) (Days 1–4, Fig 1). Baseline sleep schedules were tailored for each participant to best match their habitual schedule, based on sleep logs completed prior to the start of the study. Starting on Day 6, the sleep-wake (light-dark) schedule was shifted 9 h earlier (i.e., advanced) as though participants had flown from Chicago to Kenya, 9 time zones east (Fig 1).

During baseline and advanced days, participants remained in a bedroom/testing suite, each with their own room. Rooms were windowless, with separate, externally controlled lighting. Light levels were set to the maximum level during the first 10h of wakefulness (maximum ~ 500 lux, median 113 lux at angle of gaze) and dimmed to the lowest level (maximum < 100 lux, median 24 lux) for the last 6 h of wakefulness. Rooms were completely dark during the 8 h sleep episodes. This simulates, as much as possible in our laboratory, the experience of people who may be exposed to brighter light for the first 10 h that they are awake, and then during the 6 h after sunset (when they are still awake) are only exposed to indoor artificial lighting which is less intense. Temperature was maintained at a consistent level (73 ± 2°F or 23 ± 1°C) throughout. Participants were allowed access to cell phones, electronic devices (e.g., laptops, tablets) and time pieces (e.g., watches) during waking episodes. All devices were turned off during cognitive performance testing and were removed from rooms during sleep episodes. Each room had a wall clock indicating the ‘local’ time of either Chicago (baseline days) or Kenya (advanced days).

Meals were served at regular intervals relative to each participant’s waking time starting on day 2; 1 h (breakfast), 6 h (lunch), and 12 h (dinner) after waking. Participants were allowed up to two small snacks (≤160 calories each) per day. Beginning 2 h after waking, participants completed a test battery every 3 h (Fig 1). In this way, cognitive performance testing was also performed relative to each individual’s wake-time. There were 5 tests per day. Tests were given during the last 2 baseline days and the first 2 advanced days (Fig 1). The schedule (timing of meals and testing relative to waking time) remained the same during baseline and advanced days (Fig 1). Greater detail about cognitive performance measures are described below.

### Circadian phase

**Fig 2:**
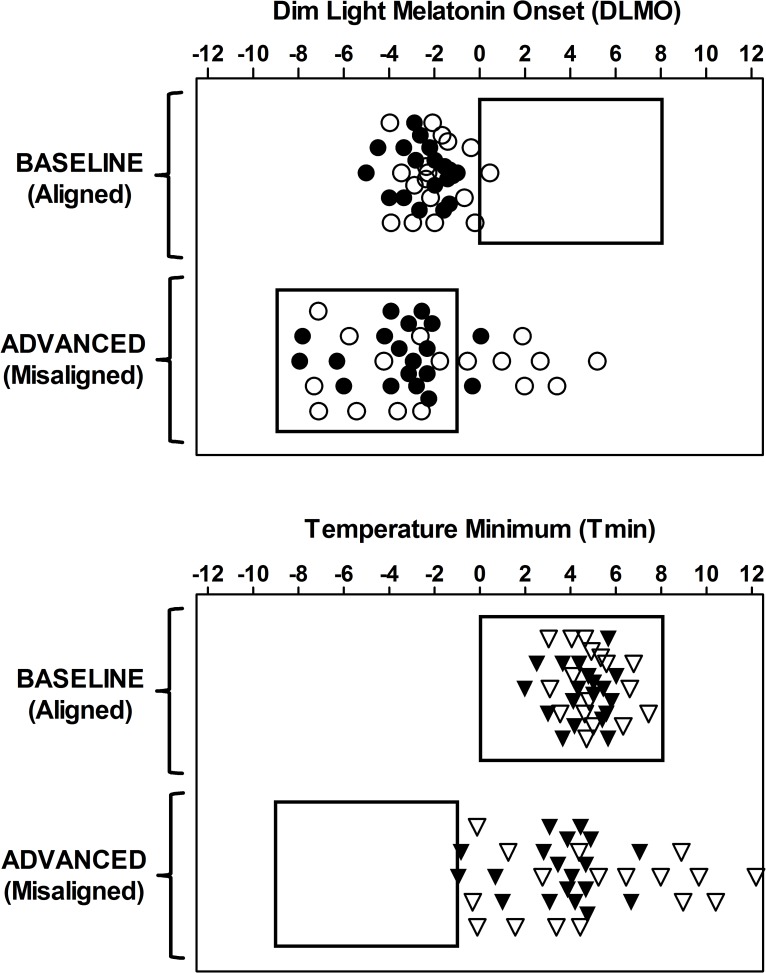
Dim light melatonin onset (DLMO) and estimated temperature minimum (Tmin) for each participant. DLMO was measured after the four baseline days on Chicago time (baseline/aligned) and after the three days on Kenya time (advanced/misaligned). Rectangles show the timing of the sleep/dark periods. Top: Circles show the DLMO relative to the baseline bedtime, with 0 representing the timing of the start of the scheduled baseline sleep period. Bottom: Triangles show the Tmin relative to baseline bedtime. The Tmin was calculated as the DLMO + 7 h. Filled symbols represent African-Americans and open symbols are European-Americans. The DLMOs and Tmins were properly aligned to the sleep/dark periods during baseline (DLMOs before sleep and Tmins within sleep), but were misaligned relative to the sleep/dark period during advanced days. The vertical symbol placement is for visualization purposes and has no relationship to days.

The method and results pertaining to circadian phase were reported in our previous publication [23]. Circadian phase markers are only presented here (Fig 2) to illustrate the enormous amount of circadian misalignment produced in this study. The method for determining circadian phase is described briefly below. On days 5 and 9 (Fig 1) participants remained seated in comfortable recliners under dim light conditions (<5 lux) during which the dim light melatonin onset (DLMO), a measure of circadian phase, was assessed. Saliva samples were collected every 30 mins using Salivettes (Starstedt, Newton, NC, USA). Samples were centrifuged, frozen, and later sent to Solid Phase Inc. (Portland Maine, USA) to be radioimmunoassayed for melatonin.[23] Based on prior work showing that the core body temperature minimum (Tmin) occurs approximately 7 h after the DLMO,[57–59] Tmin was estimated by adding 7 h to the DLMO for illustration purposes (Fig 2).

### Sleep assessment

Sleep was measured via wrist actigraphy (either Micro-Mini-Motionlogger, Ambulatory Monitoring Inc, Ardsley, New York, USA (n = 12) or Actiwatch Spectrum, Philips Respronics, Bend Oregon, USA (n = 25) and sleep diaries. Participants wore wrist activity monitors on their non-dominant wrist for the entire duration of the study. Sleep was recorded in 1-min epochs and data analyzed using either the motionlogger analysis software package (Micro-Mini-Motionlogger devices) or actiware software package (Actiwatch Spectrum devices). Due to device error, actigraphy data was lost for six participants (5 African-Americans, 1 European-American). Participants completed sleep diaries within 10 min of waking, reporting on sleep onset and offset times and any wake during the sleep episode. The primary outcome measures for both actigraphy and sleep diaries was total sleep time (TST). Actigraphy has been validated against PSG [60–62].

### Cognitive performance assessment

Participants completed the Automated Neuropsychological Assessment Metrics (ANAM) [63] test battery which was administrated on desktop computers. The ANAM test battery took approximately 20–30 min to complete and consisted of nine different assessments which were completed in the following order: subjective sleepiness, mood, simple reaction time, code substitution–learning, procedural reaction time, mathematical processing, matching to sample, code substitution–delayed (recognition), and Go/No-Go. All tasks within the ANAM battery, except the simple reaction time task, involved a set number of trials with the task ending upon completion of all trials. For tasks where a correct response was required (procedural RT, code substitution learning and delayed, mathematical processing task, and the matching to sample task) only correct responses were included in data analysis.

Subjective sleepiness was assessed using the Stanford Sleepiness Scale [64], a 7-point Likert scale where 1 = “feeling very alert, wide awake, and energetic” and 7 = “very sleepy and cannot stay awake much longer.” Participants were required to select the statement that best matched their current state. Participants rated their mood using an abbreviated 7-dimension mood scale [65], containing a set of 24 items. Using a scale of 0–6, where 0 = “not at all”, 3 (midpoint) = “somewhat”, and 6 = “very much”, participants rated each item based on their current state. Scores were grouped into seven mood dimensions; vigor (high energy level), happiness (positive disposition), depression (dysphoria), anger (negative disposition), fatigue (low energy level), anxiety (anxiety level), and restlessness (motor agitation).

Sustained attention and reaction time (RT) were measured using a 10-min simple RT task, akin to the psychomotor vigilance task (PVT) [66]. The interstimulus interval was 2–10 seconds. Participants were required to respond as quickly as possible by pressing the left mouse button to a visual stimulus (asterisk) displayed in the center of a blank screen. Lapses were defined as a RT > 500ms. Participants also completed a procedural RT task to assess processing speed and visuomotor RT when following a set of mapping rules. In this basic block version of the task, a single digit between 2 and 5 was presented in the center of the screen. Participants were required to indicate whether the number presented was ‘low’ (2 or 3) with a left mouse click, or ‘high’ (4 or 5) with a right mouse click. Slow responses (SR)–comparable to lapses–were defined as responses exceeding the 90^th^ percentile of the cumulative distribution of each participant’s baseline responses [42]. Main outcome measures for both the simple RT and the procedural RT tasks were the median RT and number of lapses or slow responses.

Two versions of the code substitution test were administered non-consecutively. The first version, code substitution–learning, was similar to the Digit Symbol Substitution Test (DSST).[67] In this test, a single digit-symbol pair was presented at the bottom of the screen. Participants were required to indicate whether the pair was correct (left mouse click) or incorrect (right mouse click) relative to a set of 9 defined digit-symbol pairs (i.e., the key) displayed at the top of the screen. Immediate feedback was provided following each response. The second version, code substitution–delayed (recognition), was identical to the first, however the key was not displayed at the top of the screen. This test was presented several minutes after the learning version, after three intervening tests. Participants were required to determine whether the displayed digit-symbol pair was correct based on the key presented earlier in the learning version. These tasks assessed sustained attention, visual scanning, associative learning and visual memory.

Basic computational skills and working memory were assessed using the mathematical processing task. In this task, participants were presented with a simple arithmetic problem (e.g., 4 + 8–5) and were required to indicate whether the answer was greater than (right mouse click) or less than (left mouse click) 5. The matching to sample task measured visuospatial working memory and processing. In this task, a pattern produced by a 4 x 4 grid with light and dark shaded cells was presented. Following a brief delay (5 sec) during which the screen was blank, two comparison grids were shown side-by-side. Participants were required to indicate with a left or right mouse click, the grid that matched the preceding grid. The primary outcome measure for the two code substitution tasks, mathematical processing, and matching to sample was the percent correct responses (number correct responses/number of trials*100).

The final task was the Go/No-Go (GNG) task, which assessed response inhibition. In this task, one of two stimuli (“x” or “o”) were presented. Participants were instructed to respond as quickly as possible to the “x” stimuli (i.e., “go”) but to do nothing, or inhibit the response (i.e., “no-go”), in response to the “o” stimuli. The number of correct responses (i.e., “hits”), incorrect responses (i.e., “false alarms”) and incorrect non-responses (i.e., “misses”) were extracted and a d-prime discriminability value was calculated (d’ = Z(hit rate)–Z(false alarm rate)). The d’ value, which was the primary outcome measure for this task, reflects the overall ability of the participant to discriminate between the go and no-go stimuli [68, 69].

Participants also completed a Columbia Jet Lag scale [70] once per day prior to each sleep episode. The jet lag scale contains a set of nine items relating to sleepiness, fatigue, daytime alertness, and concentration. Participants were required to rate how they had felt during the entire wake episode on the nine items using a scale of 0–4 where 0 = “not at all” and 4 = “extremely”. A total jet lag score was calculated from the sum of all the items.

### Data analysis

All data were analyzed using SPSS v.23 for Windows. Separate mixed model ANOVAs were performed to assess the main and interaction effects of condition (baseline or advanced) and ancestry (African-American or European-American) on sleep and cognitive performance. Regardless of main and interaction effects, additional separate mixed model ANOVAs were performed to assess the effects of ancestry on each day (sleep and Columbia Jet Lag Scale) or each hour after baseline bedtime (performance measures). These additional analyses were performed to investigate how cognitive performance varied across hours of wakefulness or across days in the study. All models were performed on all variables (sleep and cognitive performance measures). As many of these variables were nearly identical to each other, only a subset of commonly used cognitive performance measures are reported in the results. All models included participant ID as a random effect.

Where a significant difference between African-Americans and European-Americans was observed for cognitive performance measures, TST was included as a covariate to determine if this difference could be due to differences in overall sleep durations. Due to missing data from actigraphy, TST as determined from sleep logs was used. Significance was assumed at p < 0.05.

In our previous report [23] one individual (African-American) was excluded due to insufficient salivary melatonin data. This individual was included in the current study and as such, participant numbers differ between these two reports. Data on the simple RT task for one individual (African-American) was excluded as data appeared to be confounded by lack of effort as determined by a large number (> 20) of anticipatory responses (RT < 100ms).

## Results

### Circadian timing

DLMOs and Tmins were in a normal phase relationship to sleep after the four baseline days; DLMOs occurred before scheduled bedtime for all but one participant and estimated Tmins all occurred within the sleep episode (Fig 2, top panel). In contrast, after the three advanced days on Kenya time, DLMOs and Tmins for all participants were misaligned relative to the sleep episodes. None of the DLMOs occurred before sleep, and none of the Tmins occurred during sleep after the advanced days (Fig 2, bottom panel).

### Sleep

**Fig 3:**
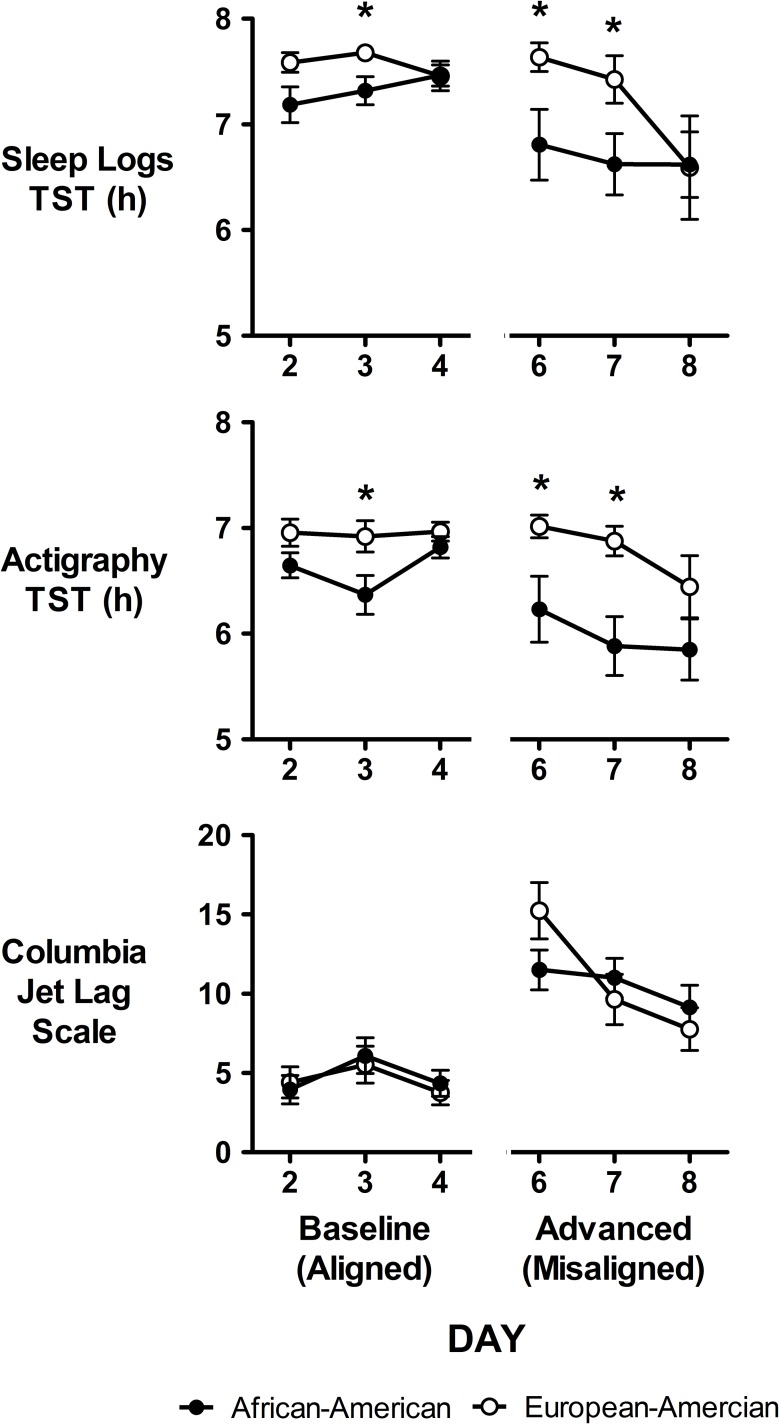
Total Sleep Time (TST) and Columbia jet lag scale scores by study day. TST was measured using sleep logs (top) and actigraphy (middle). Higher scores on the Columbia Jet Lag Scale (bottom) represent increased feelings of jet lag. Closed circles represent African-Americans and open circles represent European-Americans. N = 20 African-Americans, 17 European-Americans for sleep logs and Columbia jet lag scale. N = 15 African-Americans, 16 European-Americans for Actigraphy. Data are mean ± SEM. Baseline/aligned (days 2–4) and advanced/misaligned (days 6–8) were separated by a phase assessment day (refer to protocol, Fig 1). Asterisks denote significant differences (P ≤ 0.05) between African-Americans and European-Americans as determined by mixed model ANOVAs. There were no significant differences between African-Americans and European-Americans for Columbia Jet lag Scale scores, but there were significant differences for TST.

Fig 3 shows sleep duration for the last 3 baseline days (days 3–5) and the 3 advanced days (days 6–8). TST as measured by both actigraphy and sleep logs was significantly different between African-Americans and European-Americans and between baseline/aligned days and advanced/misaligned days (Table 2). On average, sleep was shorter during advanced/misaligned days compared to baseline/aligned days, and shorter for African-Americans compared to European-Americans (Table 3). There was a significant interaction between ancestry and condition for TST measured by actigraphy (Table 2). While TST for European-Americans did not decrease during the first two advanced/misaligned days, TST was reduced for African-Americans during all three advanced/misaligned days (Fig 3). By the third advanced day (day 8), however, there were no differences between the two groups because the TST of the European-Americans had decreased to the level of the African-Americans (Fig 3).

**Table 2:** Main and interaction effects of ancestry and condition on sleep and cognitive performance.

	Ancestry			Condition			Ancestry*Condition		
Measures	DF	F	P	DF	F	P	DF	F	P
TST Logs	1,35	4.08	0.05*	1,183	15.17	0.00*	1,183	1.21	0.27
TST Actigraphy	1,29	8.58	0.01*	1,150	18.67	0.00*	1,150	6.67	0.01*
Columbia Jet Lag Scale	1,35	0.00	0.97	1,183	105.27	0.00*	1,183	0.23	0.63
SSS	1,35	0.22	0.64	1,701	72.69	0.00*	1,701	0.01	0.95
SRT Lapses	1,34	3.55	0.07	1,682	0.02	0.90	1,682	0.10	0.75
SRT Median RT	1,34	2.80	0.10	1,682	0.02	0.88	1,682	0.36	0.55
CSL % correct	1,35	0.00	0.97	1,701	9.88	0.00*	1,701	2.32	0.13
CSD % correct	1,35	1.95	0.17	1,701	6.78	0.01*	1,701	2.85	0.09
GNG d-prime	1,35	0.84	0.37	1,701	7.95	0.01*	1,701	0.01	0.95
MTH % correct	1,35	2.05	0.16	1,701	0.98	0.32	1,701	0.73	0.39
M2S % correct	1,35	0.17	0.68	1,701	1.78	0.18	1,701	0.15	0.70
Pro RT SR	1,35	3.04	0.09	1,701	2.47	0.12	1,701	5.56	0.02*
Pro RT Median RT	1,35	0.83	0.37	1,701	1.26	0.26	1,701	2.61	0.11
Mood—Vigor	1,35	0.58	0.45	1,701	74.31	0.00*	1,701	1.61	0.21
Mood- Happiness	1,35	0.27	0.61	1,701	48.38	0.00*	1,701	8.31	0.00*
Mood- Depression	1,35	2.51	0.12	1,701	0.12	0.73	1,701	0.29	0.89
Mood- Anger	1,35	1.34	0.26	1,701	0.04	0.84	1,701	4.06	0.04*
Mood- Fatigue	1,35	0.01	0.94	1,701	53.98	0.00*	1,701	1.15	0.29
Mood- Anxiety	1,35	0.06	0.80	1,701	10.93	0.00*	1,701	3.37	0.07
Mood- Restlessness	1,35	0.40	0.53	1,701	0.06	0.81	1,701	1.44	0.23

Ancestry was either African-American or European-American and Condition was either Baseline (circadian rhythms aligned with sleep) or Advanced (circadian rhythms misaligned relative to sleep).

Significance was assumed at P≤0.05 as indicated by an asterisk.

TST: Total Sleep Time. SSS: Stanford Sleepiness Scale. SRT: Simple Reaction Time, lapses (RT <500ms.). CSL % correct: Code Substitution-Learning percent correct responses. CSD % correct: Code Substitution-Delayed percent correct responses. GNG d-Prime: Go/No-Go task d-prime score. MTH % correct: Mathematical Processing task percent correct responses. M2S % correct: Matching to Sample task percent correct responses. Pro RT SR: Procedural Reaction Time task number of Slow Responses (responses exceeding the 90^th^ percentile of the cumulative distribution of each participant’s baseline responses). Pro RT Median RT: Procedural Reaction Time task median RT. For the SRT task, data from one participants was excluded and some actigraphy data was lost due to device malfunction (refer to methods).

**Table 3:** Means for sleep and cognitive performance measures for ancestry and condition.

	Ancestry		Condition	
Measure	African- American	European- American	Baseline (Aligned)	Advanced (Misaligned)
TST Logs	7.00 ± 1.11	7.40 ± 1.03 ^a^	7.44 ± 0.55	6.93 ± 1.39 ^b^
TST Actigraphy	6.30 ± 0.93	6.87 ± 0.65 ^a^	6.79 ± 0.54	6.40 ± 1.04 ^b^^,^^c^
Jet Lag Scale	7.68 ± 5.86	7.73 ± 6.58	4.69 ± 4.18	10.70 ± 6.39 ^b^
SSS	2.95 ± 1.49	2.83 ± 1.34	2.53 ± 1.24	3.27 ± 1.50 ^b^
SRT Lapses	6.81 ± 8.81	3.63 ± 5.20	5.29 ± 7.59	5.33 ± 7.40
SRT Medium RT	323.34 ± 60.57	296.89 ± 45.48	310.98 ± 57.14	310.72 ± 53.94
CSL % correct	97.09 ± 3.39	97.11 ± 3.14	97.41 ± 2.72	96.79 ± 3.72 ^b^
CSD % correct	74.85 ± 18.76	81.56 ± 18.51	79.18 ± 17.95	76.69 ± 19.81^b^
GNG d-prime	2.95 ± 0.92	2.71 ± 1.11	2.91 ± 0.91	2.77 ± 1.12 ^b^
MTH % correct	93.16 ± 8.68	95.53 ± 6.28	94.49 ± 6.78	94.01 ± 8.63
M2S % correct	93.33 ± 8.08	94.10 ± 8.55	94.00 ± 7.27	93.36 ± 9.23
Pro RT SR	3.49 ± 2.95	2.98 ± 2.21	3.09 ± 2.06	3.42 ± 3.11
Pro RT Median RT	510.58 ± 78.82	492.07 ± 60.19	500.28 ± 71.97	503.87 ± 70.94 ^c^
Mood—Vigor	2.09 ± 1.28	2.34 ± 1.16	2.43 ± 1.23	1.97 ± 1.19 ^b^
Mood- Happiness	3.25 ± 1.39	3.45 ± 1.27	3.51 ± 1.26	3.17 ± 1.39 ^b^^,^^c^
Mood- Depression	0.12 ± 0.36	0.35 ± 0.79	0.22 ± 0.63	0.23 ± 0.58
Mood- Anger	0.27 ± 0.59	0.48 ± 0.87	0.37 ± 0.72	0.37 ± 0.76 ^c^
Mood- Fatigue	1.54 ± 1.21	1.51 ± 1.43	1.28 ± 1.18	1.77 ± 1.40 ^b^
Mood- Anxiety	0.31 ± 0.53	0.35 ± 0.73	0.28 ± 0.61	0.37 ± 0.64 ^b^
Mood- Restlessness	0.81 ± 0.98	0.98 ± 1.08	0.89 ± 1.00	0.88 ± 1.07

Values shown as mean ± SD.

^a^ significantly different from African-Americans

^b^ significantly different from baseline/aligned

^c^ Significant interaction between ancestry and condition

TST: Total Sleep Time. SSS: Stanford Sleepiness Scale. SRT Simple Reaction Time task, lapses (RT <500ms.).CSL % correct: Code Substitution-Learning percent correct responses. CSD % correct: Code Substitution-Delayed percent correct responses. GNG d-Prime: Go/No-Go task d-prime score. MTH % correct: Mathematical Processing task percent correct responses. M2S % correct: Matching to Sample task percent correct responses. Pro RT SR: Procedural Reaction Time task number of Slow Responses (responses exceeding the 90^th^ percentile of the cumulative distribution of each participant’s baseline responses). Pro RT Median RT: Procedural Reaction Time task median RT. Mood: seven mood sub-scales; vigor, happiness, depression, anger, fatigue, anxiety, and restlessness. Higher scores indicate worse cognitive performance for all measures except code substitution (learning and delayed) and Mathematical Processing, where higher scores indicate better cognitive performance.

Columbia Jet Lag Scale scores were significantly higher during advanced/misaligned days compared to baseline; however, there were no differences between African-Americans and European-Americans (Tables 2 and 3 and Fig 3).

### Cognitive performance

**Fig 4:**
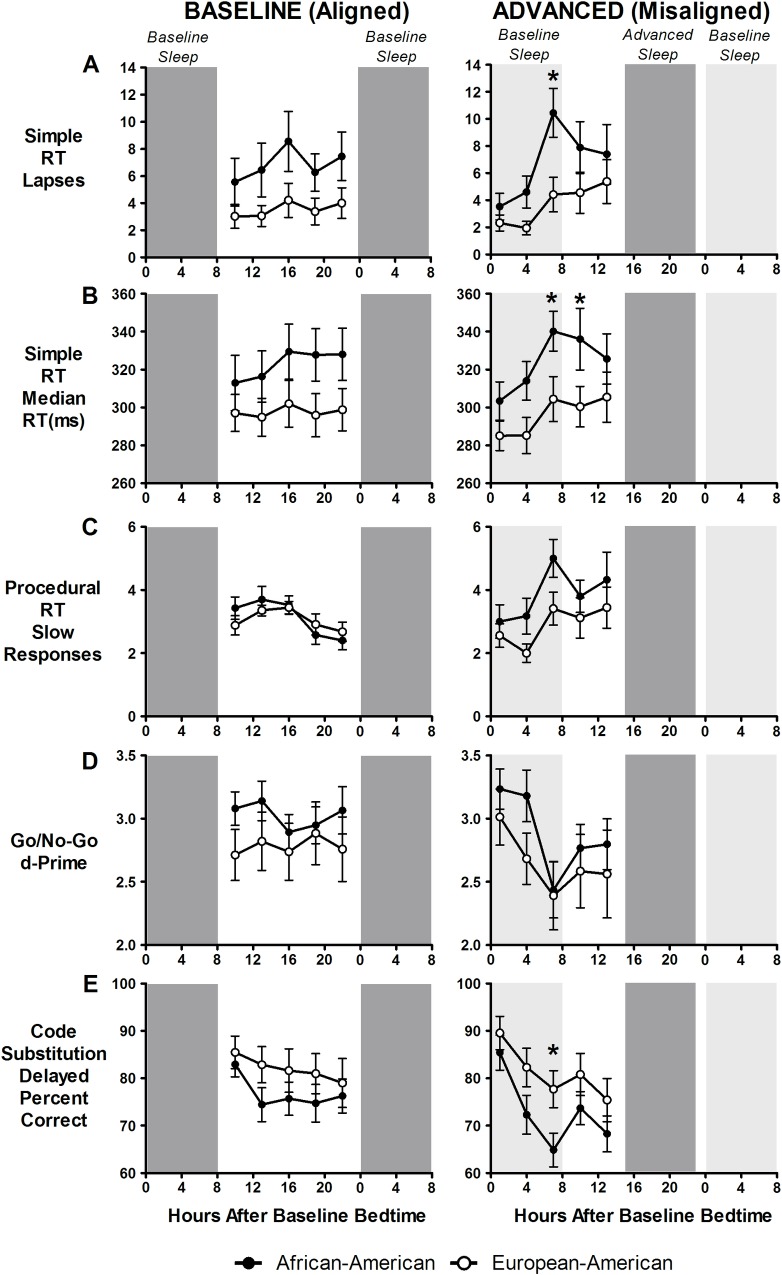
Cognitive performance during baseline/aligned and advanced/misaligned days. Performance was assessed on the simple reaction (RT) time task (A and B), procedural RT task (C), Go/No-Go task (D), and code substitution-delayed task (E). Closed circles represent African-Americans and open circles represent European-Americans. Dark grey shading represents timing of scheduled sleep/dark episodes during both baseline/aligned and advanced/misaligned days. Light grey shading on the right panels represents the previous baseline sleep episode. The first three cognitive performance tests (1, 4 and 7-h after baseline bedtime) during the advanced/misaligned days occurred when participants would normally be sleeping (i.e. during the scheduled baseline sleep/dark episode). Lapses (A) were defined as being RTs < 500ms, slow responses (C) were responses exceeding the 90^th^ percentile of the cumulative distribution of each participant’s baseline responses, and d-Prime scores (D) were the discriminability values indicating the overall ability of to discriminate between the go and no-go stimuli. Higher scores for A, B, and C represent worse cognitive performance. Lower scores for D and E represent worse cognitive performance. N = 19 African-Americans and 17 European-Americans (A and B). N = 20 African-Americans and 17 European-Americans (C, D and E). Asterisks denote significant differences (P ≤ 0.05) between African-Americans and European-Americans at each hour after baseline bedtime as determined by mixed model ANOVAs. There were no statistically significant differences between African-Americans and European-Americans during baseline/aligned, but there were differences found during advanced/misaligned days (right panels), particularly at the time corresponding to the end of baseline sleep.

**Fig 5:**
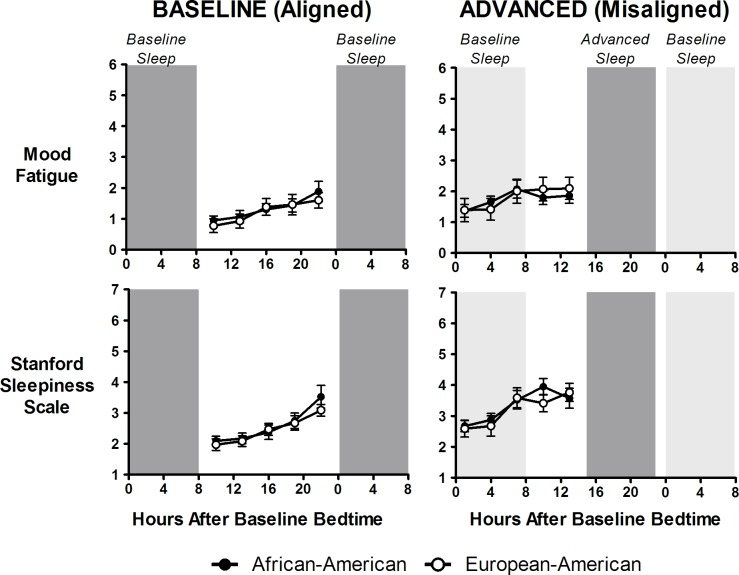
Subjective sleepiness and fatigue (low energy level) during baseline/aligned and advanced/misaligned days. Closed circles represent African-Americans and open circles represent European-Americans. Dark grey shading represents timing of scheduled sleep/dark episodes during both baseline and advanced days. Light grey shading on right panels (advanced) represents the previous baseline sleep/dark episodes. Top panel shows subjective ratings on the fatigue mood dimension (low energy level) and the bottom panel shows subjective sleepiness (Stanford Sleepiness Scale). Fatigue scores were on a scale of 0–6 and the Stanford Sleepiness Scale is a scale of 1–7. For both measures higher scores represent higher feelings of fatigue/sleepiness. N = 20 African-Americans and 17 European-Americans. Sleepiness and fatigue were higher during advanced/misaligned days compared to baseline/aligned days, but there were no significant differences between African-Americans and European-Americans for either measure at any time point.

Fig 4 shows cognitive performance outcomes and Fig 5 shows subjective sleepiness and fatigue. During baseline, there were no significant differences between African-Americans and European-Americans in cognitive performance on the simple RT task (lapses and median RT), procedural RT (slow responses), Go/No-Go task (d-prime), or code substitution-delayed (Fig 4, left panels). When the schedule was advanced/misaligned, cognitive performance worsened, particularly around 7-h after baseline bedtime (Fig 4, right panels), corresponding to the end of the scheduled baseline sleep episode. After that there was a slight improvement in cognitive performance. This worsening of cognitive performance tended to be more pronounced in African-Americans on the simple RT and code substitution-delayed tasks. Specifically, there was a significant difference found between the two groups 7-h after baseline bedtime on the simple RT (lapses and median RT) and code substitution, and 10-h after baseline bedtime on the median RT (Fig 4, right panels). When TST was applied as a covariate, only the code substitution–delayed remained significantly different between ancestry groups (Fig 4), suggesting that the difference between African-Americans and European-Americans during advanced/misaligned days on the simple RT may be explained by differences in TST.

There were no significant main effects of ancestry on any of the cognitive performance measures or subjective scales; however, there were significant main effects of condition for several measures including subjective sleepiness (SSS), code substitution (learning and delayed), Go/No-Go and mood (vigor, happiness, fatigue and anxiety) (Table 2). Cognitive performance, subjective ratings of sleepiness, and mood worsened during advanced/misaligned days compared to baseline (Table 3). When TST was covaried, many of these significant differences remained, suggesting that sleep duration was not explaining this effect (S1 Table).

There was a significant interaction for Procedural RT (slow responses) (Table 2). Cognitive performance was similar between baseline/aligned and advanced/misaligned days for European-Americans. However, during advanced/misaligned days, cognitive performance was worse for African-Americans compared to baseline (Fig 4). There were also significant interaction effects for the mood dimensions of happiness and anger (Table 2).

Subjective reports of sleepiness (SSS) and the fatigue (low energy level) mood dimension showed a gradual increase with increasing hours after baseline bedtime (i.e., increasing hours of wakefulness). Sleepiness and fatigue were greater during advanced/misaligned days, but there were no differences between African-Americans and European-Americans during either baseline/aligned or advanced/misaligned days (Fig 5).

## Discussion

The current study is, to our knowledge, the first investigating how a large abrupt phase shift in the sleep/wake (and light/dark) schedule, similar to that observed with jet travel across many time zones and many shiftwork schedules, affects the sleep and cognitive performance of African-Americans compared to European-Americans. During baseline days, when the sleep/wake schedule was aligned with the endogenous circadian rhythms, there was little difference in sleep and no differences in cognitive performance between African-Americans and European-Americans. During advanced days, however, when the sleep/wake schedule was greatly misaligned with the endogenous circadian rhythms, African-Americans slept significantly less and there was a trend for them to perform worse compared to European-Americans.

Some of these results were unexpected, as we had hypothesized that African-Americans would sleep more and perform better compared to European-Americans after an advance in the sleep/wake schedule, as they have shorter free-running circadian periods which should facilitate adjusting to phase advances [23, 24]. In retrospect, these results make sense because neither ancestry group phase shifted enough in the first two days after the shift in the sleep/wake schedule (and light-dark cycle) to adjust to the changed schedule. Our definition and goal for circadian adaptation is to phase shift the circadian clock enough to bring the temperature minimum into the new time for sleep [71]. Fig 2 showed that none of the estimated temperature minima occurred during the advanced sleep episodes, even after three days on the advanced schedule. Perhaps if we had continued the experiment with more days on the advanced schedule, then the expected differences between the ancestry groups may have emerged. The current results show what happens during extreme circadian misalignment.

In concordance with previous findings [9–15] in the current study, on average, African-Americans obtained less sleep than European-Americans. Here we showed that the shorter sleep of African-Americans was further shortened by an advance in the sleep/wake schedule. While some studies have previously suggested that differences in sleep duration may be due to socio-economic status (SES), environmental and social factors [6, 7], results from the current study do not support this. SES did not differ between the African-Americans and European-Americans in the current study. Previous work has shown that when SES is controlled for, either by matching the population or via statistical analyses, differences in sleep duration between African-Americans and European-Americans remain [4, 9, 11, 12, 14, 72]. Together, the current study and these previous reports would suggest that it is more likely that biological, mostly likely genetic, differences are the primary drivers of differences in sleep duration between people with different evolutionary ancestries.

In the current study, as demonstrated in Fig 2, part of the advanced sleep episode occurred during the ‘wake maintenance zone’ [73] or the ‘forbidden zone for sleep’ [74], an interval of a few hours occurring shortly before bedtime (around the DLMO) that is associated with a strong circadian drive for wakefulness and subsequently reduced sleep [19–22, 31]. While this explains the reduced sleep experienced by African-Americans, European-Americans obtained the same amount of sleep on the first two days following the advance as they did during baseline days. The advance in the sleep/wake schedule occurred following a circadian phase assessment during which participants were required to be awake for a total of 31h (Fig 1). This extended wakefulness likely increased the homeostatic pressure for sleep (i.e., increased the pressure for sleep). There is some evidence suggesting that under conditions of sleep loss, the increased homeostatic pressure for sleep may mask the circadian drive for wakefulness [75]. These results would imply that European-Americans accumulated more homeostatic pressure than African-Americans during the extended wakefulness, such that this increased homeostatic pressure masked the circadian drive for wakefulness, maintaining TST at near baseline levels.

Indeed, there is evidence that there are differences in the expression of slow wave sleep (SWS) and slow wave activity (SWA) between ethnic/ancestry groups [9, 10, 14–17]. These measures are considered to be markers of sleep pressure [76, 77]. African-Americans show less SWS and SWA than European-Americans [9, 10, 14–17], with a higher percentage of African-American genetic ancestry directly associated with a decrease in SWS [10]. Taken together, previous findings suggest that African-Americans may accumulate less homeostatic pressure during wakefulness than European-Americans. Although we did not measure waking electroencephalography (EEG), and therefore do not have a marker of homeostatic pressure, results from the current study suggest that European-Americans may accumulate a higher homeostatic pressure than African-American, with this higher homeostatic pressure masking the circadian drive for wakefulness when the sleep episode was advanced. As to why there would be evolutionary differences in the homeostatic and circadian regulation of sleep is unknown and should be investigated further.

During the advanced/misaligned days, in concordance with previous work [31–34], cognitive performance exhibited a circadian influence. With increasing wakefulness cognitive performance worsened, peaking around 7 h after baseline bedtime, before improving after the end of the baseline bedtime (i.e., during the baseline waking hours). Although this circadian influence appeared for both African-Americans and European-Americans, cognitive performance impairments tended to be more prominent in African-Americans. During the baseline days, cognitive performance was similar between African-Americans and European-Americans, suggesting that ancestry does not directly influence cognitive performance. Under conditions of sleep loss, the circadian influence on cognitive performance is exacerbated [35–37], suggesting that the trend for additional cognitive performance impairments observed in African-Americans compared to European-Americans following the advance in the sleep/wake schedule may be due, at least in part, to the reduced sleep experienced by African-Americans.

Simple cognitive tasks such as the simple RT task, akin to the PVT, are susceptible to the effects of sleep loss [78, 79]. More complex tasks, however, such as the Procedural RT or Go/No-Go, may require multiple cognitive processes and appear to be less susceptible to the effects of sleep loss [78, 79]. The differences in vulnerability of cognitive performance measures to sleep loss depends on the complexity of the task and may explain why African-Americans performed worse than European-Americans on some tasks (e.g., simple RT), while there were no differences for more complex tasks (e.g., Go/No-Go). It is important to note however, that for many of the tasks where we observed a significant difference between African-Americans and European-Americans during the advanced/misaligned days (Fig 4, right column), there was no significant interaction effect between condition and ancestry (Table 2). For other tasks, we observed a significant interaction effect, but there were no significant differences between African-Americans and European-Americans during the advanced/misaligned days. This suggests that these findings may be statistical irregularities and further research is needed to confirm the trend for African-Americans to have poorer cognitive performance than European-Americans following an advance in the sleep/wake schedule.

Following the advance in the sleep/wake schedule, during misaligned days, participants reported increased feelings of sleepiness on the SSS and some mood disturbances such as increased fatigue (low energy levels) and anxiety, and decreased happiness and vigor. While there were changes to mood during misaligned days, differences in scores were low, suggesting that the impact was mild while living in a laboratory. Further, although African-Americans reported worsened mood during misaligned days compared to European-Americans, the difference in scores were also small and are of little clinical significance [65]. An increase in subjective sleepiness and fatigue during misaligned days was expected due to the known effects of sleep loss and circadian misalignment on subjective ratings [30, 31, 64, 80, 81]. The absence of differences in subjective ratings between African-Americans and European-Americans, despite there being differences in cognitive performance during misaligned days, highlights the lack of congruence between subjective measures and objective cognitive performance measures [30, 36, 81], and shows the need for objective measures of cognitive performance.

The current study suggests that advancing the sleep/wake schedule, has differential effects on the sleep and cognitive performance of African-Americans and European-Americans. However, there are some limitations that should be considered. Participants in the current study were young, healthy adults who remained in a controlled laboratory environment and therefore these results may not be directly applicable to the wider population. There was a large amount of variability in several of our measures, particularly cognitive performance measures, which may have reduced the overall statistical power of the study. This may have been avoided if power analyses were performed for the cognitive performance and sleep measures, which is one limitation of these analyses. Of note is that power analyses were performed for the main measures reported in the paper describing circadian period and phase shifts [23]. Despite this, these results still provide preliminary evidence that there may be ancestry differences in sleep and cognitive performance in response to an abrupt shift in the sleep/wake schedule.

In the current study, sleep was measured using actigraphy. Although actigraphy has been shown to correlate well with PSG–the gold standard measure of sleep–it may not always be able to accurately differentiate between periods of still wakefulness and sleep [60–62]. Further, actigraphy is unable to distinguish between individual sleep stages, such as SWS. As such, it is not possible to determine from current results if the homeostatic and circadian influences on sleep differ between African-Americans and European-Americans, it is only possible to speculate. Further research should include measures of PSG to gain a better understanding of how the circadian and homeostatic influences may differ depending on ancestry.

A key advantage the current study, however, was the genetic assessment of ancestry. While most previous studies relied on self-identification of race, which may incorporate geographical ancestry, physical traits such as skin tone, as well as social factors such as culture and history [82], the current study used public accessible genetic testing to confirm ancestry in addition to asking participants about the ethnicities/races of their biological parents. This genetic testing revealed that one individual, who had self-identified as being African-American, had almost equal parts African and European ancestries, highlighting the importance of including an objective measure of ancestry in addition to self-assessments [83].

Current results suggest that advancing the sleep/wake schedule such that sleep and wake become misaligned relative to the endogenous circadian rhythms–similar to what is observed with jet travel and shift work–affects the sleep of African-Americans more than the of European-Americans. In African-Americans, the additional sleep loss may contribute to the greater cognitive performance impairment than that experienced by European-Americans. Although sleep disruption and cognitive performance impairments have consequences for both African-Americans and European-Americans, results may be particularly critical for African-Americans, who are more likely to work shift work schedules compared to European-Americans [84]. Findings have implications for how African-Americans may respond to jet travel or shiftwork relative to European-Americans.

## Supporting information

S1 Appendix (PDF)

S2 Appendix (PDF)

S3 Appendix (PDF)

S1 Excel Data File (XLSX)

S1 Table Main and interaction effects of ancestry and condition on cognitive performance measures with total sleep time included as a covariate. Total Sleep Time from sleep logs was included in mixed model analyses as a covariate. Ancestry was either African-American or European-American and Condition was either baseline/aligned or advanced/misaligned. Significance was assumed at P≤0.05 as indicated by an asterisk. SSS: Stanford Sleepiness Scale. SRT: Simple Reaction Time task, lapses (RT < 500ms.). CSL % correct: Code Substitution-Learning percent correct responses. CSD % correct: Code Substitution-Delayed percent correct responses. GNG d-Prime: Go/No-Go task d-prime score. MTH % correct: Mathematical Processing task percent correct responses. M2S % correct: Matching to Sample task percent correct responses. Pro RT SR: Procedural Reaction Time task number of Slow Responses (responses exceeding the 90^th^ percentile of the cumulative distribution of each participant’s baseline responses). Pro RT Median RT: Procedural Reaction Time task median RT.
(DOCX)
