# Supplementary material for: Advancing the sleep/wake schedule impacts the sleep of African-Americans more than European-Americans
Source: PLoS One. 2017 Oct 23;12(10):e0186887. doi: 10.1371/journal.pone.0186887 (PMC5653363; doi:10.1371/journal.pone.0186887)
Supplement: S1 Appendix — (PDF) [file pone.0186887.s001.pdf]

FAMILY / ANCESTOR FORM

Biological Rhythms Research Lab
Rush University Medical Center

Name ____ Date __________________
Month / Day / Year

We are interested in learning about the body clock (circadian clock) of people from all the different ethnic and racial backgrounds. In order to make sure we have people from a variety of different ethnic and racial groups, we need to ask you some questions about your background. We will also be asking questions about the race/ethnicity of your parents and grandparents. Feel free to call your relatives for help. If necessary, you will be able to take this form home to get more information.

1. Where were you born?

A. City ____ Don't know _________

B. State ____ Don't know _________

C. Country ____ Don't know _________

2. Please put check marks by your race/ethnicity (check **ALL** that apply).
Please circle the word or words in the descriptions below if they led you to choose any of the categories.

A. WHITE ______
A person whose family and ancestors came from Europe, the Middle East or North Africa.

B. BLACK OR AFRICAN AMERICAN _______
A person whose family and ancestors belonged to any of the black racial groups of Africa.

C. ASIAN ______
A person whose family and ancestors came from the Far East, Southeast Asia, or the Indian subcontinent, including, for example, Cambodia, China, India, Japan, Korea, Malaysia, Pakistan, the Philippine Islands, Thailand, and Vietnam.

D. HISPANIC OR LATINO ________
A person of Cuban, Mexican, Puerto Rican, South or Central American, or other Spanish culture or origin, regardless of "race."

E. EUROPEAN _______
A person whose family and ancestors came from Britain, Ireland, France, Spain, Sweden, Germany, Poland, Russia, Yugoslavia, Romania, Lithuania, etc.

F. MIDDLE EASTERN ______
A person whose family and ancestors came from Saudi Arabia, Iraq, Iran, Turkey, Syria, Jordan, Lebanon, Israel, etc. Also those who consider themselves Arabian, Persian, etc.

(SEE MORE CATEGORIES ON THE NEXT PAGE)

G. FAR EAST ASIAN ___________
A person whose family and ancestors came from China, Japan, Korea, Hong Kong, Taiwan, etc.

H. SOUTHEAST ASIAN _________
A person whose family and ancestors came from Indonesia, Thailand, Cambodia, Malaysia, the Philippines, Singapore, Laos, Vietnam, Burma (Myanmar), etc.

I. INDIAN SUBCONTINENT __________
A person whose family and ancestors came from India, Afghanistan, Pakistan, Bangladesh, Nepal, Sri Lanka, etc.

J. NORTH AFRICAN ______
A person whose family and ancestors came from Algeria, Morocco, Egypt, Libya, Tunisia, etc.

K. AFRO-CARIBBEAN ______
A person whose family and ancestors came from Jamaica, Haiti, the Dominican Republic, etc. Also, those who consider themselves Creole.

L. AMERICAN INDIAN OR ALASKA NATIVE ______
A person whose family and ancestors came from North, Central, or South America.

M. NATIVE HAWAIIAN OR OTHER PACIFIC ISLANDER ______
A person whose family and ancestors came from Hawaii, Guam, Samoa, or other Pacific Islands.

N. OTHER____________
If possible please elaborate. If you think there are additional or better terms to describe your race/ethnicity please add them here.

____

____

____

____

____

O. DON'T KNOW ________________

On the next pages please fill in as much as you can about your blood relatives.

IF YOU ARE ADOPTED and do not know anything about your biological parents check here and skip the rest of this form. _____________

YOUR BIOLOGICAL MOTHER

1. Please fill in her place of birth.

A. City ____ Don't know _________

B. State _______________________________________ Don't know _________

C. Country _____________________________________ Don't know _________

2. Please put check marks for her race/ethnicity (check **ALL** that apply).
Look back at the first 2 pages for definitions. Please choose all of the words from those definitions that apply to her and write them below.

A. WHITE ____

B. BLACK OR AFRICAN AMERICAN ___________________________________

C. ASIAN ____

D. HISPANIC OR LATINO ____

E. EUROPEAN ____

F. MIDDLE EASTERN ____

G. FAR EAST ASIAN ____

H. SOUTHEAST ASIAN ____

I. INDIAN SUBCONTINENT ____

J. NORTH AFRICAN ____

K. AFRO-CARIBBEAN ____

L. AMERICAN INDIAN OR ALASKA NATIVE _______________________________

M. NATIVE HAWAIIAN OR OTHER PACIFIC ISLANDER _______________________

N. OTHER____________
If possible please elaborate. If you think there are additional or better terms to describe her race/ethnicity please add them here.

____

____

____

O. DON'T KNOW ________________

YOUR BIOLOGICAL FATHER

1. Please fill in his place of birth.

A. City ____ Don't know _________

B. State ____ Don't know _________

C. Country ____ Don't know _________

2. Please put check marks for his race/ethnicity (check **ALL** that apply).
Look back at the first 2 pages for definitions. Please choose all of the words from those definitions that apply to him and write them below.

A. WHITE ____

B. BLACK OR AFRICAN AMERICAN ____

C. ASIAN ____

D. HISPANIC OR LATINO ____

E. EUROPEAN ____

F. MIDDLE EASTERN ____

G. FAR EAST ASIAN ____

H. SOUTHEAST ASIAN ____

I. INDIAN SUBCONTINENT ____

J. NORTH AFRICAN ____

K. AFRO-CARIBBEAN ____

L. AMERICAN INDIAN OR ALASKA NATIVE ___________________________________

M. NATIVE HAWAIIAN OR OTHER PACIFIC ISLANDER ________________________

N. OTHER____________
If possible please elaborate. If you think there are additional or better terms to describe his race/ethnicity please add them here.

____

____

____

O. DON'T KNOW ________________

YOUR GRANDMOTHER ON YOUR MOTHER'S SIDE
(YOUR MOTHER'S BIOLOGICAL MOTHER)

1. Please fill in her place of birth.

A. City ____ Don't know _________

B. State _______________________________________ Don't know _________

C. Country _____________________________________ Don't know _________

2. Please put check marks for her race/ethnicity (check **ALL** that apply).
Look back at the first 2 pages for definitions. Please choose all of the words from those definitions that apply to her and write them below.

A. WHITE ____

B. BLACK OR AFRICAN AMERICAN _____________________________________

C. ASIAN ____

D. HISPANIC OR LATINO ____

E. EUROPEAN ____

F. MIDDLE EASTERN ____

G. FAR EAST ASIAN ____

H. SOUTHEAST ASIAN ____

I. INDIAN SUBCONTINENT ____

J. NORTH AFRICAN ____

K. AFRO-CARIBBEAN ____

L. AMERICAN INDIAN OR ALASKA NATIVE ________________________________

M. NATIVE HAWAIIAN OR OTHER PACIFIC ISLANDER ______________________

N. OTHER____________
If possible please elaborate. If you think there are additional or better terms to describe her race/ethnicity please add them here.

____

____

____

O. DON'T KNOW ________________

YOUR GRANDFATHER ON YOUR MOTHER'S SIDE
(YOUR MOTHERS BIOLOGICAL FATHER)

1. Please fill in his place of birth.

A. City ____ Don't know _________

B. State ____ Don't know _________

C. Country ____ Don't know _________

2. Please put check marks for his race/ethnicity (check **ALL** that apply).
Look back at the first 2 pages for definitions. Please choose all of the words from those definitions that apply to him and write them below.

A. WHITE ____

B. BLACK OR AFRICAN AMERICAN ____

C. ASIAN ____

D. HISPANIC OR LATINO ____

E. EUROPEAN ____

F. MIDDLE EASTERN ____

G. FAR EAST ASIAN ____

H. SOUTHEAST ASIAN ____

I. INDIAN SUBCONTINENT ____

J. NORTH AFRICAN ____

K. AFRO-CARIBBEAN ____

L. AMERICAN INDIAN OR ALASKA NATIVE ___________________________________

M. NATIVE HAWAIIAN OR OTHER PACIFIC ISLANDER ________________________

N. OTHER____________
If possible please elaborate. If you think there are additional or better terms to describe his race/ethnicity please add them here.

____

____

____

O. DON'T KNOW ________________

YOUR GRANDMOTHER ON YOUR FATHER'S SIDE
(YOUR FATHER'S BIOLOGICAL MOTHER)

1. Please fill in her place of birth.

A. City ____ Don't know _________

B. State ____ Don't know _________

C. Country _______________________________________ Don't know _________

2. Please put check marks for her race/ethnicity (check **ALL** that apply).
Look back at the first 2 pages for definitions. Please choose all of the words from those definitions that apply to her and write them below.

A. WHITE ____

B. BLACK OR AFRICAN AMERICAN _______________________________________

C. ASIAN ____

D. HISPANIC OR LATINO ____

E. EUROPEAN ____

F. MIDDLE EASTERN ____

G. FAR EAST ASIAN ____

H. SOUTHEAST ASIAN ____

I. INDIAN SUBCONTINENT ____

J. NORTH AFRICAN ____

K. AFRO-CARIBBEAN ____

L. AMERICAN INDIAN OR ALASKA NATIVE _________________________________

M. NATIVE HAWAIIAN OR OTHER PACIFIC ISLANDER _______________________

N. OTHER____________
If possible please elaborate. If you think there are additional or better terms to describe her race/ethnicity please add them here.

____

____

____

O. DON'T KNOW ________________

YOUR GRANDFATHER ON YOUR FATHER'S SIDE
(YOUR FATHER'S BIOLOGICAL FATHER)

1. Please fill in his place of birth.

A. City ____ Don't know _________

B. State ____ Don't know _________

C. Country _______________________________________ Don't know _________

2. Please put check marks for his race/ethnicity (check **ALL** that apply).
Look back at the first 2 pages for definitions. Please choose all of the words from those definitions that apply to him and write them below.

A. WHITE ____

B. BLACK OR AFRICAN AMERICAN _______________________________________

C. ASIAN ____

D. HISPANIC OR LATINO ____

E. EUROPEAN ____

F. MIDDLE EASTERN ____

G. FAR EAST ASIAN ____

H. SOUTHEAST ASIAN ____

I. INDIAN SUBCONTINENT ____

J. NORTH AFRICAN ____

K. AFRO-CARIBBEAN ____

L. AMERICAN INDIAN OR ALASKA NATIVE _________________________________

M. NATIVE HAWAIIAN OR OTHER PACIFIC ISLANDER _______________________

N. OTHER____________
If possible please elaborate. If you think there are additional or better terms to describe his race/ethnicity please add them here.

____

____

____

O. DON'T KNOW ________________
