# Supplementary material for: Advancing the sleep/wake schedule impacts the sleep of African-Americans more than European-Americans
Source: PLoS One. 2017 Oct 23;12(10):e0186887. doi: 10.1371/journal.pone.0186887 (PMC5653363; doi:10.1371/journal.pone.0186887)
Supplement: S2 Appendix — (PDF) [file pone.0186887.s002.pdf]

Name: ______________________________________ Date: _________________

HEALTH INFORMATION QUESTIONNAIRE

1. In general, how would you describe your health?

   Excellent………………………… 1
   Good ………………………...2
   Fair ………………………...3
   Poor ………………………...4

2. During the past 6 months, have you been hospitalized for any reason?

   Yes………………………………1
   No……………………………….. 2

   A. If "YES," how many times?_____ Times

   B. For a TOTAL of how long? _____ Days

   C. Why were you hospitalized? ____

3. During the past month, about how many days of sick leave did you take? _____ Days
   (PLEASE WRITE "0" IF NONE)

4. PLEASE INDICATE ABOUT HOW OFTEN YOU USED EACH OF THE FOLLOWING MEDICATIONS DURING THE PAST MONTH:

	Medications	Not at all	Less than once a a week	1-2 times a week	3-4 times a week	Every day
a.	Aspirin or headache medicine	1	2	3	4	5
b.	Aids for stomach or digestion problems	1	2	3	4	5
c.	Laxatives	1	2	3	4	5
d.	Cough, cold or sinus medicine	1	2	3	4	5
e.	Medicine to pep you up	1	2	3	4	5
f.	Medication to calm you down	1	2	3	4	5
g.	Prescription medicine:					
	1. ______________________________ (Please specifiy)		2	3	4	5
	2. ______________________________ (Please specifiy)		2	3	4	5
	3. ______________________________ (Please specifiy)		2	3	4	5

5. THE FOLLOWING QUESTIONS CONCERN YOUR BODY FUNCTIONS. PLEASE TRY TO ANSWER EACH QUESTION BY CIRCLING A NUMBER TO INDICATE HOW OFTEN YOU HAVE EXPERIENCED EACH OF THE FOLLOWING ITEMS WITHIN THE PAST YEAR.

	Never	Occasionally	Frequently	Constantly
1. Intense, irresistible urge to move your legs along with numbness, tingling, pricking, burning, itching, and/or electric shock……………………………	1	2	3	4
2. Uncomfortable sensations in your legs that become worse when resting……………………………..……	1	2	3	4
3. Uncomfortable sensations in your legs that are partially, or totally, relieved by movement…………….……..…..	1	2	3	4
4. Increased severity of uncomfortable sensations in your legs in the evening or at night …………………..	1	2	3	4
5. Motor restlessness, unable to stay still…………..........	1	2	3	4
6. Shortness of breath or trouble breathing………………..	1	2	3	4
7. Frequent colds or sore throats…………………………..	1	2	3	4
8. Persistent cough and spitting up sputum………………..	1	2	3	4
9. Coughing up blood…………………………………...	1	2	3	4
10. Fever, chills, and aching all over………………………..	1	2	3	4
11. Hay fever or sinus trouble……………………………….	1	2	3	4
12. Wheezing in your chest………………………………….	1	2	3	4
13. Jaundice, yellow eyes or skin………………………….	1	2	3	4
14. Itching skin, skin rash, allergic skin reactions………..	1	2	3	4
15. Swollen or painful muscles and joints…………………	1	2	3	4
16. Back pain……………………………………………	1	2	3	4
17. Pain or stiffness in your arms or legs………………….	1	2	3	4
18. Tearing or itching of eyes……………………………..	1	2	3	4
19. Persistent numbness or tingling in any part of your body…………………………………………….…	1	2	3	4
20. Ringing or buzzing in ears…………………………..	1	2	3	4
21. Severe headaches…………………………………..…	1	2	3	4
22. Fainting spells or dizziness…………………………….	1	2	3	4
23. Nervous or shaking inside…………………………...	1	2	3	4
24. Times when you feel sweaty or trembly………………	1	2	3	4
25. Increased urination……………………………………..	1	2	3	4
26. Painful urination……………………………………..	1	2	3	4
27. Bloody urine……………………………………………	1	2	3	4

	Never	Occasionally	Frequently	Constantly
28. Alarming pain or pressure in your chest……………….	1	2	3	4
29. Pain down your arms………………………………...	1	2	3	4
30. "Racing" or pounding heart…………………….….	1	2	3	4
31. Leg cramps………………………………………..	1	2	3	4
32. Periods of severe fatigue or exhaustion………………...	1	2	3	4
33. Acid indigestion, heartburn, or acid stomach…………..	1	2	3	4
34. Diarrhea for more than a few days……………………..	1	2	3	4
35. Gas or gas pains……………………………………...	1	2	3	4
36. Nausea or vomiting…………………………………...	1	2	3	4
37. Blood in your bowel movement………………………..	1	2	3	4
38. Constipation…………………………………………….	1	2	3	4
39. Tight feeling in stomach………………………………..	1	2	3	4
40. Bloated or full feeling…………………………………..	1	2	3	4
41. Feeling of pressure in the neck………………………….	1	2	3	4
42. Hemorrhoids or piles……………………………………	1	2	3	4
43. Trouble digesting food………………………………….	1	2	3	4
44. Blurred vision…………………………………………...	1	2	3	4
45. Dryness in the mouth……………………………………	1	2	3	4
46. Bleeding gums when brushing teeth…………………...	1	2	3	4
47. Stomach pains…………………………………………...	1	2	3	4
48. Belching…………………………………………………	1	2	3	4
49. Difficulty with feet and legs when standing for long periods……………………………………………..	1	2	3	4

6. Within the past 5 years has a doctor ever treated your for, or told you that you had: (please circle)

	No	Yes	(If yes, give approximate date when symptoms first appeared)	Don't know
1. Diabetes………………………………………………	1	2	____/____ Year / Mo.	3
2. Cancer………………………………………………..	1	2	____/____ Year / Mo.	3
3. Hernia or rupture……………………………………..	1	2	____/____ Year / Mo.	3
4. Tuberculosis………………………………………….	1	2	____/____ Year / Mo.	3
5. Asthma……………………………………………….	1	2	____/____ Year / Mo.	3

6. "High" blood pressure………………….………….	1	2	____/____ Year / Mo.	3
7. Heart disease………………………………………	1	2	____/____ Year / Mo.	3
8. Arthritis…..……………………………………………	1	2	____/____ Year / Mo.	3
9. Epilepsy….………………………………………...	1	2	____/____ Year / Mo.	3
10. Glaucoma of the eye……………………………….	1	2	____/____ Year / Mo.	3
11. Paralysis, tremor, or shaking………………………	1	2	____/____ Year / Mo.	3
12. Kidney or bladder trouble…………………………….	1	2	____/____ Year / Mo.	3
13. Lung or breathing problems…………………………..	1	2	____/____ Year / Mo.	3
14. Stroke…………………………………………………	1	2	____/____ Year / Mo.	3
15. Anemia……………………………………………..	1	2	____/____ Year / Mo.	3
16. Gall bladder, liver, or pancreas trouble……………….	1	2	____/____ Year / Mo.	3
17. Thyroid trouble or goiter……………………………...	1	2	____/____ Year / Mo.	3
18. Insomnia……………………………………………	1	2	____/____ Year / Mo.	3
19. Gastritis…………………………………………….	1	2	____/____ Year / Mo.	3
20. Colitus…………………………………………………	1	2	____/____ Year / Mo.	3
21. Stomach ulcer………………………………………	1	2	____/____ Year / Mo.	3

7. During a typical week, on approximately how many days do you drink alcoholic beverages? _____Days
(Mark 0 if you do not drink alcoholic beverages)

A. If you drink, do you ever drink on those days when you work?
Yes…….……………………………1
No…………………………………...2

B. IF "YES": Do you normally drink…(Circle all that apply)
Before going to work……………… 1
After finishing work………………. 2
During lunch breaks………………. 3
During rest breaks when at work…. 4

8. On those days when you do drink, about how many of each of the following do you usually drink:
(Mark 0 if you do not drink alcoholic beverages)

a. Bottles of beer_____ Bottles
b. Glasses of wine____ Glasses
c. Shots of liquor (shot = 1½ oz.) ____ Shots

9. Would you say that the amount of alcohol you have been drinking late has…

   Increased……..……………………….. 1
   Decreased…...………………………2
   Remained about the same…………. 3
   Don't drink…………………...……. 4

10. Would you say that you use alcohol to help you get to sleep?

   Frequently….………………………. 1
   Occasionally..………………………2
   Seldom……………….…………. 3
   Never……..…………………...…….4

11. On an average day, how many of each of the following do you smoke? (Mark 0 if you do NOT smoke)

   a. Cigarettes _____Cigarettes
   b. Cigars _____Cigars
   c. Pipefuls of tobacco _____Pipefuls
