# Supplementary material for: Advancing the sleep/wake schedule impacts the sleep of African-Americans more than European-Americans
Source: PLoS One. 2017 Oct 23;12(10):e0186887. doi: 10.1371/journal.pone.0186887 (PMC5653363; doi:10.1371/journal.pone.0186887)
Supplement: S3 Appendix — (PDF) [file pone.0186887.s003.pdf]

FOR WOMEN ONLY

The purpose of this questionnaire is to give us information about your menstrual cycle, which may affect your levels of melatonin.

Name ____ Date ______________________

1. Have you ever had menstrual periods?

   Yes _____ No _____

   If NO, then thank you, you don't have to answer any more questions.

2. Have you ever had surgery that caused your periods to stop permanently (such as removal of your ovaries)?

   Yes _____ No _____

   If YES, then thank you, you don't have to answer any more questions.

3. Have you ever experienced unusual breast discharge?

   Yes _____ No _____

   If YES, then thank you, you don't have to answer any more questions.

4. Are you pregnant? Yes _____ No _____

   If YES, then thank you, you don't have to answer any more questions.

5. Are you trying to get pregnant? Yes _____ No _____

   If YES, then thank you, you don't have to answer any more questions.

6. Have you ever tried to get pregnant without success?

   Yes _____ No _____

7. Have you ever been pregnant?

   Yes _____ No _____

   If YES, how long ago? _____________

   (If less than 12 months ago, then thank you, you don't have to answer any more questions).

8. Are you currently breast feeding?

   Yes _____ No _____

   If YES, then thank you, you don't have to answer any more questions.

9. Are you taking birth control pills? Yes _____ No _____(if NO, skip to question #10).

   If YES, Name of prescription ____

   Date you started the last box of pills ______________________________________

   Day of week you start your pills ____

   For how many months or years have you been taking birth control pills? __________________

   Please skip to **question #13.**

10. Have you taken birth control pills in the past? Yes _____ No _____

    If YES, When did you stop using them? ______________________________________

11. Are your menstrual cycles regular (i.e. can you usually predict the date of your next period)?

    Yes _____ No _____

    Comments: ____

    ____

    ____

12. What is the average length of your menstrual cycle (i.e. the number of days between the <u>start</u> of one period and the <u>start</u> of the next period. 28 days is a typical length)?

    ____

13. When did your last period start? Date ___________________ Day of Week ___________________

14. If you have kept track of the dates of your menstrual periods, please list the last 5 here:

    ____

    ____

    ____

15. Are you taking any other medications that contain hormones (e.g. hormonal patch, injections, or fertility drugs)? Yes _____ No _____

    Please provide details____

16. Are you taking any nutritional supplements or alternative medications?

Yes _____ No _____

If YES, then please list them:____

17. Have you ever had a tubaligation (had your "tubes tied")?

Yes _____ No _____

If YES, then when did you have it done? __________________________

18. The following questions are for women over 40 years old. If you are younger than 40, then please skip to question #25. If you are over 40 years, please go to question #19 and complete the entire questionnaire.

19. Are you taking a contraceptive?

Yes _____ No _____

If YES, then thank you, you don't have to answer any more questions.

If NO, then please go to question #20.

20. Do you or your doctor think that

a) you are going through menopause.

b) you are menopausal.

c) Neither.

21. Are you taking supplemental or replacement hormones for menopause or symptoms of menopause, such as hot flashes? Yes _____ Yes, but I stopped _____ No, never took them _____

22. Have you noticed any irregularity in your menstrual periods, such as the following:

Bleeding between your periods	Yes _____ No _____Maybe______
A change in the length of your periods	Yes _____ No _____Maybe______
A change in the interval between your periods	Yes _____ No _____Maybe______
A change in the heaviness of your periods	Yes _____ No _____Maybe______

23. In your family, at what age did your mother, aunts and other close female relatives have menopause?

______________ years ________don't know

24. Have you noticed an unusual physical or emotional changes recently (such as crying spells, depression)?

Yes _____ No _____Maybe______

If YES please provide details____

25. Do you usually feel bad in the week before your period
(sometimes called PMS, or premenstrual syndrome)?
Yes _____ No _____Maybe______

Please check any of the following that you usually notice in the week before your period:

- ❑ Feel depressed, hopeless, worthless or guilty
- ❑ Feel anxious or tense
- ❑ Have mood swings or your feelings are easily hurt
- ❑ Feel angry or irritable
- ❑ Have less interest in work, school, friends or hobbies
- ❑ Have difficulty concentrating
- ❑ Feel tired
- ❑ Have an increased appetite or overeating
- ❑ Sleep more or less than usual
- ❑ Feel overwhelmed and unable to cope
- ❑ Have breast tenderness, weight gain, or headache

Do any of these symptoms:

A. Interfere with your productivity at work, school, home or in your daily routine?

Yes _____ No _____Maybe______

B. Cause you to avoid or participate less in hobbies or social activities?

Yes _____ No _____Maybe______

C. Interfere with your relationships with others?

Yes _____ No _____Maybe______
