# Supplementary material for: Advancing the sleep/wake schedule impacts the sleep of African-Americans more than European-Americans
Source: PLoS One. 2017 Oct 23;12(10):e0186887. doi: 10.1371/journal.pone.0186887 (PMC5653363; doi:10.1371/journal.pone.0186887)
Supplement: S1 Table — Total Sleep Time from sleep logs was included in mixed model analyses as a covariate. Ancestry was either African-American or European-American and Condition was either baseline/aligned or advanced/misaligned. Significance was assumed at P≤0.05 as indicated by an asterisk. SSS: Stanford Sleepiness Scale. SRT: Simple Reaction Time task, lapses (RT < 500ms.). CSL % correct: Code Substitution-Learning percent correct responses. CSD % correct: Code Substitution-Delayed percent correct responses. GNG d-Prime: Go/No-Go task d-prime score. MTH % correct: Mathematical Processing task percent correct responses. M2S % correct: Matching to Sample task percent correct responses. Pro RT SR: Procedural Reaction Time task number of Slow Responses (responses exceeding the 90th percentile of the cumulative distribution of each participant’s baseline responses). Pro RT Median RT: Procedural Reaction Time task median RT. (DOCX) [file pone.0186887.s005.docx]

**S1 Table. Main and interaction effects of Ancestry and Condition on performance measures with Total Sleep Time included as a covariate.**

|  | Ancestry | |  | Condition | |  | Ancestry*Condition | |
| --- | --- | --- | --- | --- | --- | --- | --- | --- |
| Measures | F | P |  | F | P |  | F | P |
| SSS | 0.00 | 0.97 |  | 58.11 | 0.00* |  | 0.37 | 0.54 |
| SRT Lapses | 2.73 | 0.11 |  | 1.15 | 0.23 |  | 2.14 | 0.14 |
| SRT Median | 1.74 | 0.20 |  | 5.93 | 0.02* |  | 2.23 | 0.14 |
| CSL % correct | 0.02 | 0.90 |  | 10.42 | 0.00* |  | 2.61 | 0.11 |
| CSD % correct | 1.75 | 0.19 |  | 5.52 | 0.02* |  | 2.36 | 0.13 |
| GNG d-prime | 1.14 | 0.29 |  | 5.53 | 0.02* |  | 0.05 | 0.83 |
| MTH % correct | 2.30 | 0.14 |  | 1.38 | 0.24 |  | 0.98 | 0.32 |
| M2S % correct | 0.21 | 0.65 |  | 1.93 | 0.17 |  | 0.20 | 0.66 |
| Pro RT SR | 1.12 | 0.30 |  | 0.98 | 0.32 |  | 3.85 | 0.05 |
| Pro RT Median RT | 0.61 | 0.44 |  | 0.41 | 0.53 |  | 1.64 | 0.20 |
| Mood - Vigor | 0.22 | 0.64 |  | 57.33 | 0.00* |  | 0.37 | 0.54 |
| Mood- Happiness | 0.17 | 0.68 |  | 40.81 | 0.00* |  | 6.58 | 0.01* |
| Mood- Depression | 2.51 | 0.12 |  | 0.11 | 0.74 |  | 0.27 | 0.60 |
| Mood- Anger | 1.89 | 0.18 |  | 0.55 | 0.46 |  | 2.64 | 0.11 |
| Mood- Fatigue | 0.14 | 0.71 |  | 38.09 | 0.00* |  | 3.49 | 0.06 |
| Mood- Anxiety | 0.11 | 0.75 |  | 8.87 | 0.00* |  | 2.70 | 0.10 |
| Mood- Restlessness | 0.70 | 0.41 |  | 0.79 | 0.37 |  | 0.55 | 0.46 |

Total Sleep Time from sleep logs was included in mixed model analyses as a covariate. Ancestry was either African-American or European-American and Condition was either baseline (aligned) or advanced (misaligned). Significance was assumed at P≤0.05 as indicated by an asterisk. SSS: Stanford Sleepiness Scale. SRT: Simple Reaction Time task, lapses (RT < 500ms.). CSL % correct: Code Substitution-Learning percent correct responses. CSD % correct: Code Substitution-Delayed percent correct responses. GNG d-Prime: Go/No-Go task d-prime score. MTH % correct: Mathematical Processing task percent correct responses. M2S % correct: Matching to Sample task percent correct responses. Pro RT SR: Procedural Reaction Time task number of Slow Responses (responses exceeding the 90^th^ percentile of the cumulative distribution of each participant’s baseline responses). Pro RT Median RT: Procedural Reaction Time task median RT.
